# Venous Nanoflap Oscillations: Biomechanical Determinants and Hydrodynamic Consequences in the Deep Cerebral Venous System

**DOI:** 10.3390/ijms27125202

**Published:** 2026-06-09

**Authors:** Raluca Florentina Tulin, Stefan Oprea, Mihaly Enyedi, Adrian Vasile Dumitru, Dan Dumitrescu

**Affiliations:** 1 Faculty of General Medicine, “Carol Davila” University of Medicine and Pharmacy, 050474 Bucharest, Romaniadan.dumitrescu@umfcd.ro (D.D.); 2 Department of Anatomy, “Carol Davila” University of Medicine and Pharmacy, 050474 Bucharest, Romania; 3 Department of Pathology, Faculty of Medicine, “Carol Davila” University of Medicine and Pharmacy, 030167 Bucharest, Romania; 4 Puls Med Association, 051885 Bucharest, Romania; 5 Department of General Surgery, “Carol Davila” University of Medicine and Pharmacy, 050474 Bucharest, Romania

**Keywords:** nanomechanical venous oscillation, endothelial nanoflaps, neurovascular biomechanics, nanoscale hydrodynamics, resonance drift, lipid lattice viscoelasticity, mechanotransductive signaling, clearance microenvironments, oscillatory instability

## Abstract

The most recent research has demonstrated that oscillatory nano-structures found on the lumenal walls of deep cerebral veins likely contribute significantly to the regulation of the function of deep cerebral veins. The oscillatory nano-structures consist of very small, intricately organized “nanoflaps,” each consisting of a hinge element with an attached lipid bilayer architecture. These nanoflaps have distinct mechanical properties, are in close proximity to mechanically sensitive protein assemblies, and therefore it is hypothesized that the nanoflaps generate rhythmic oscillations that control the distribution of both pressure and fluid flow through the veins and also regulate the metabolic condition of the surrounding tissue. In addition, the behavior of the nanoflaps indicate that there exists a hitherto unappreciated level of venous biomechanics at the nanometer scale that regulates the hydraulic stability of the veins and may also contribute to the structural integrity of the surrounding tissues. The purpose of this review is to provide a theoretical framework for understanding the recent discoveries of the structure, oscillation and hydrodynamic effects of nanoflaps, including resonance drift, waveform irregularity, and multi-scale biomechanical interactions. Additionally, this review will present the idea that disruption of the normal oscillatory processes that occur in the nanoflaps may lead to the development of abnormal micro-environments in the early stages of neurodegenerative diseases, abnormalities of compliance, dysautonomic states, traumatic injury and micro-circulatory stress. Finally, this review will describe several pharmacological strategies that may be used to stabilize the oscillations generated by the nanometer-scale oscillatory nano-structure by modifying the torque applied to the hinge, the viscoelasticity of the membrane and the feedback pathways for mechanotransduction.

## 1. Introduction

It was previously believed that the cerebral venous system could be best understood using a macroscopic approach where cerebral veins were seen as low pressure capacitance vessels draining blood from the brain based upon overall pressure gradients, luminal diameter and vessel compliance [1]. While this model still serves as the foundation for understanding the cerebral venous system it has also been shaped by imaging modalities that have limited ability to resolve the endothelial lumen and therefore cannot fully utilize the fine scale architectural features of the venous system to describe the flow characteristics of the system. Therefore, cerebral veins have historically been viewed as generally smooth walled vessels with simple architectural geometries and thus primarily influenced by parameters of large vessel hemodynamics [2]. High resolution vascular imaging along with nanoscale structural evaluation now suggests that this view of cerebral veins may be overly simplistic. Recent findings have shown that the endothelial lining of major cerebral veins contains distinct membrane protrusions that measure roughly 200–900 nm in length and 20–70 nm in width [3]. The majority of these protrusions were found in areas of the brain where deep venous drainage occurs and therefore the flow within the venous system may be subjected to greater hydrodynamic forces [4,5]. Furthermore, unlike typical static anatomical irregularities; these protrusions demonstrate oscillatory motion that varies depending on local hemodynamic conditions. It has been hypothesized that the mechanism for this oscillatory motion is due to flexible, hinge like attachments to the underlying endothelial cortex allowing for bending and twisting motions in response to minor changes in wall shear stress [6,7]. Using high frame rate analyses, researchers have demonstrated that these protrusions exhibit oscillatory frequencies ranging from 40–120 Hz with harmonics exceeding 200 Hz under conditions of increased pulsatility [8].

The observed behavior is similar to that of nanometer-scale biologic oscillators, which operate outside of the spatial domain examined in traditional vascular physiology. Therefore, if nanometer-scale oscillating structures exist, their influence on hemodynamics may exceed their physical dimensions. Studies using near wall flow modeling and micro-fluidic analog systems have shown that nanometer-scale oscillatory movements may alter the local velocity gradient of the boundary layer and create micro-scaled vortex flow regimes resulting in transient retrograde pressure waves [9,10]. The time required for the creation of these micro-flow phenomena is estimated to occur over a period measured in milliseconds (typically 10–50 ms), which depends upon several variables including oscillatory magnitude, wall shear rates and geometry of the venous segment. However, when considering the curved geometry of many deep venous siphon segments, even slight disturbances created by localized flow separation, brief transient backward flow and short lived stagnant zones, can significantly alter the longitudinal flow profile without apparent occlusion or stenosis [11,12].

This consideration is particularly important since the deep cerebral venous system directly interacts with perivascular clearance pathways including cerebrospinal fluid-interstitium exchange and glymphatic transport. Computational and experimental investigations indicate that pressure wave disturbances as small as ~0.1–0.3 mm Hg can decouple phase relationships between venous pulsatility, CSF motion and interstitial convective velocities [13]. Furthermore, increased amplitude of oscillatory activity has been correlated with decreased net clearance efficiency, redirection of interstitial flow and generation of low velocity perivascular retention zones containing accumulated metabolites proximal to segments exhibiting unstable venous flow. These types of retention mechanisms are likely to contribute to various pathological states characterized by disrupted glymphatic function, including white matter degeneration, clearance failure during sleep and post-traumatic disruption of ICP regulation [14,15].

From a molecular standpoint, there appears to be compatibility between proposed oscillatory behaviors of putative venous nanoflaps and established principles governing cellular mechanobiology. Actin—spectrin-based cortical assemblies and Ezrin-Radixin- Moesin ERM family proteins involved in linkage between membrane-cytoskeleton networks are likely candidates providing mechanical support to the hinge-like regions of these nanoflaps while lipid domains rich in sphingomyelin and cholesterol will determine the anisotropic flexibility of lamellae-shaped regions of the nanoflapse [16,17]. Moreover, rapidly responding mechanically sensitive ion channels (PIEZO1, TRPV4 etc.) embedded within the membrane will allow for instantaneous feedback responses between membrane deformations and subsequent cytoskeletal rearrangements [18]. Thus, whereas nanoflap motion represents a locally active form of mechanotransduction capable of modulating its oscillatory characteristics dynamically; it does so independently of being simply a passive mechanical phenomenon. Collectively, these lines of evidence establish that deep cerebral venous flow may be subject to influences across different spatial scales. Mechanisms of nanoscale membrane mechanics can potentially influence hydrodynamical processes occurring at micrometer spatial scales which can further impact millimeter-scale clearance mechanisms influencing physiological homeostasis at tissue levels [19]. From this perspective, the deep venous system can be considered as a multi-scale biomechanical environment where early disruptions may originate not from collapse, inflammation or gross stenosis of the vein itself but from very subtle alterations in resonance characteristics at the endothelial interface [20,21].

The goal of this review is not to present definitive evidence for venous nanoflap biology but instead provide a theoretical framework for organizing existing data relating to the morphology, oscillatory characteristics and potential pathophysiological impacts of venous nanoflap dynamics in concert with known paradigms of mechanobiology, membrane physics and neurovascular fluid dynamics. Therefore, this review integrates all relevant information currently available regarding venous nanoflap morphology, oscillatory characteristics and potential pathophysiological effects and provides insights into future therapeutic interventions aimed at stabilizing these nanometer-scale mechanical systems in order to promote effective clearance and maintain hydraulic homeostasis in the brain.

## 2. The Venous Nanoflap: Structural, Topological, and Biophysical Identity

### 2.1. Ultra-Resolved Structural Architecture and Nanoscale Spatial Organization

Ultra-high-resolution imaging has made possible increasingly accurate and detailed visualizations of thin-walled lumenular membrane architecture along deep cerebrovenous surfaces. In contrast to earlier interpretations of ultra-thin membrane “protrusions” (nanoflap-like), this form of architecture can be viewed as multicomponent biomechanical assemblies whose internal structure is organized reproducibly [22]. Recent imaging studies have identified what appears to be a tripartite structural arrangement of the biomechanical assembly. The base of the assembly is connected to the endothelial cortex via a reinforced hinge area. The central area of the assembly is a flexible lamellar portion that generates the majority of the oscillating movement. A tapering fin-like distal end portion of the assembly appears particularly sensitive to curvature and local perturbations in flow. Interferometric contour maps of the hinge region show that typically the width of the hinge will vary between 80 and 140 nm. Angular excursion ranges are typically less than 20°. Although the narrowness of the angular excursion range provides some degree of mechanical constraint against excessive repeated deformation, restricts the rotational freedom of the assembly, and may provide additional structural integrity when subjected to high frequency oscillatory loads; the hinge does not appear to completely constrain flapping movements. Instead, it acts as an actively controlled pivoting mechanism which seeks to balance flexibility with stability of position [23]. As indicated above, the lamellar component of the nanoflaphemically contains alternately dense and sparse membrane platelets, approximately 8–15 nm thick [24].

These two types of layers have differing bending moduli and lateral compressibilities. As such, they would produce a stratified flexural system capable of storing and redistributing oscillation energy in response to small amplitude motions. Additionally, this multi-layered nature may enhance resistance to fatigue due to repeated high frequency loading and allow repeated deformation without sudden structural failure. Nanoflap like components appear to be distributed throughout quasi-periodic groups within deep midline venous territories. Center-to-center spacing was measured to be in the range of 1.2–1.7 µm [25], and the grouping appears to be preferentially located near areas of increased intrinsic wall curvature. Distribution patterns indicate that nanoflap formation may be influenced by heterogeneous membrane tension in preference over a random distribution along the venous surface. More importantly, while nearest neighbor relationships appear to be significant, long-range periodicity is limited, indicating that positioning of nanoflaps may result from a process of localized mechanical self-organization within the endothelial cortex, as opposed to a global predetermined developmental pattern [26].

Rotational analysis of three dimensional space indicates that there exists some degree of torsional preload along the longitudinal axis of the nanoflap, estimated to be in the range of 1.8–5.4°/100 nm. Torsional preload may cause slight deviation of nanoflap position either laterally or circumferentially during oscillation causing redistribution of mechanical stresses through the structure. This could assist in reducing localized stress concentrations, enable elastic recovery following transient deformation, and assist in providing nanoflap structural resiliency under various shear conditions. Therefore, based on these findings we interpret nanoflaps as being composed of specialized, spatially regulated biomechanical structures exhibiting distinct architectural logic [27].

### 2.2. Biochemical and Biophysical Composition: Determinants of Hinge Elasticity and Lamellar Mechanics

The structural integrity of the hinge area of the nanoflaps is therefore most likely provided by membrane-cytoskeleton linkage proteins that mechanically link the cell membrane to the cortical cytoskeleton. Instead of referring to generic “linker” proteins, specific potential molecules include those of the ERM family along with the βII-spectrin-actin junctional complexes; both types of molecules have been shown to provide structural support to curved membranes and regulate cortical tension, and both can transduce mechanical forces generated at the cell surface to the cell cortex [28]. In this configuration, the hinge would represent a compact force transmitting domain where ERM enriched connection points and spectrin supported filament networks represent a mechanically responsive pivot architecture. Force probe experiments indicate that the torque stiffness of this hinge-like area is in the order of 10^−3^ to 10^−2^ N·m^−1^·rad^−1^, under physiologic ionic conditions, providing adequate resistance to structural damage upon repeated cyclic loading and providing enough compliance to allow for rapid rotational movement in response to shear. Forces greater than about 30 pN are thought to cause conformational changes in the cytoskeletal assemblies associated with the hinge; such changes are believed to result from the mechanical disruption of phospho-regulated actin linked membrane/cortical complexes indicating that the hinge is a dynamic structure capable of mechanically regulated restructuring [29,30].

It appears that the lamellar component of the nanoflap is primarily made up of liquid-ordered membrane domains exhibiting lateral asymmetry in lipid composition. Nanosampled lipidomics data indicates there are gradients in the ratios of saturated to unsaturated lipids present in different regions of the lamella, suggesting there will be variations in lateral compressibility and bending modulus throughout the lamella [31]. Specifically, it is hypothesized that the higher concentrations of sphingomyelin and cholesterol in ordered microdomains closer to the base of the lamella will create stiffening effects at those locations creating stable bending patterns, whereas less rigid regions located farther from the base of the lamella may exhibit increased flexibility under shear-induced loading. This gradient pattern supports the concept that the lamella represents a heterogeneously elastic rather than uniformly elastic membrane sheet. Cholesterol nanoclusters approximating diameters of 6–14 nm could provide additional stabilization by acting as discrete anchoring points for limiting excessive lateral stretching of the lamella and maintaining consistent bending periodicities [32]; thus supporting wave fidelity during oscillation and aiding in maintaining structural cohesiveness under repeated dynamic stresses. A second critical factor influencing nanoflap behavior includes a large number of mechanosensitive ion channels found at the hinge-lamella interface. While direct identification of molecular specificity remains a requirement, candidate mechanosensitive ion channel families for this application include PIEZO1, TRPV4, and possibly ASIC mechanosensitive/mechanomodulated channels; each of which have previously demonstrated involvement in endothelial responses to membrane tension, curvature, and shear stress [33]. It is also logical to describe these channels as generating high sensitivity signaling microdomains with approximate densities ranging from 90–150 channels per μm^2^; thus permitting rapid detection of local mechanical disruptions. Upon activation by deformation of the lamella, these channels are predicted to produce localized Ca^2+^ influx events lasting less than approximately 12 milliseconds, and extending over distances less than 400 nm from their point(s) of initiation [34].

Localized Ca^2+^ microtransients produced by these channels are functionally significant since they can rapidly alter local actomyosin contractility, ERM phosphorylation, and membrane-cortex associations resulting in changes in hinge stiffness on time scales approaching seconds. Therefore, mechanical deformation can be converted into biochemical signals through a mechanochemically based feedback mechanism allowing the nanoflap to adapt its mechanics in real-time to changes in shear stress and pulsatile loads [35]. Therefore, the venous nanoflap can be viewed as a composite biomechanical system consisting of: (1) ERM-spectrin-actin hinge complexes, (2) anisotropically structured lipid micro-domains, and (3) mechanosensitive ion-channel signal-processing units centered on PIEZO1/TRPV4. The collective molecular organization described here provides a more detailed explanation for the oscillatory behavior described above, and supports a view of the nanoflap as a structurally mechanosensitive endothelial unit rather than simply a passive membraneous irregularity [36].

### 2.3. Oscillatory Regimes, Resonant Behaviors, and Nonlinear Dynamical Transitions

Nanoflaps vibrate in a wide variety of dynamic states based upon the level of shear stress, flow pulsatility, viscosity gradients and the local geometry of the vessel. At lower levels of shear stress, the nanoflap exists in a mechanically quiescent state with little to no displacement (less than 5 nanometers) and little to no baseline frequency oscillation at frequencies ranging from 15 to 25 Hz. The mechanically quiescent state is energetically ideal and does not cause significant disturbance to the adjacent boundary layer [37,38].

At intermediate flow accelerations, the nanoflap transitions into a basic resonant vibration mode. The frequency of the vibrations is generally between 40 and 90 Hz. The amplitude of the vibrations is generally between 20 and 40 nanometers. During this regime of vibration, the vibrations follow a smooth sinusoidal path and create localized, spatially-constrained distortions to the velocity profile of the lumen [39].

Upon exposure to higher levels of shear stress or rapid increases in pressure gradient, the nanoflap transitions into either a second or third harmonic vibration mode. The frequency of the harmonic modes is generally 2.1 times and 3.3 times the frequency of the fundamental resonant vibration mode [40]. The harmonic modes of vibration are generally non-symmetric. The harmonic modes of vibration often include delayed posterior inflection points along the lamella and minor longitudinal phase gradients. The degree of asymmetry of the harmonic modes of vibration is significantly related to micro-vortex generation. Therefore, there is evidence to support the hypothesis that the harmonic vibrations of the flaps play a mechanistic role in initiating turbulence at the micro-scale [41,42].

**Figure 1:**
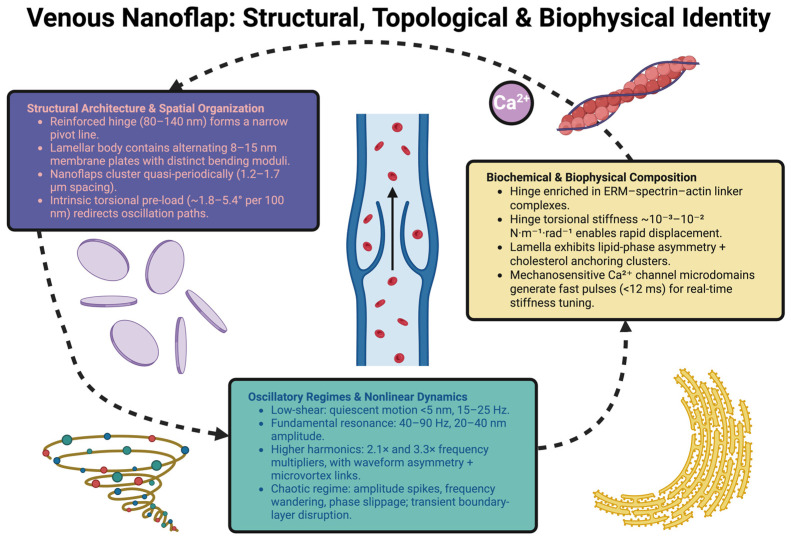
Conceptual overview of the proposed venous nanoflap and its structural, biophysical, and dynamic organization. The schematic summarizes three integrated aspects of the proposed venous nanoflap: (i) structural architecture and spatial organization, including a narrow hinge region, lamellar body, and quasi-periodic clustering along the venous wall; (ii) biochemical and biophysical composition, including spectrin–actin/ERM-associated hinge complexes, lipid-phase heterogeneity, and mechanosensitive Ca^2+^-related microdomains; and (iii) oscillatory behavior, ranging from low-amplitude motion to higher-frequency and potentially irregular regimes under increased hemodynamic stress. The figure is intended as a conceptual synthesis of experimentally grounded and model-informed features, rather than a direct anatomical depiction.

This Figure 1 aims to summarize the structural, biochemical, and dynamical principles that define the venous nanoflap as a specialized biomechanical organelle rather than a passive surface irregularity.

Upon experience of fluctuations in intracranial pulsatility or sudden changes in compliance of the surrounding parenchyma, the nanoflap can enter a rapid transition into a chaotic dynamical state. Chaotic vibrations are characterized by random amplitude spikes, frequency wander and phase slip. The vibrations are highly sensitive to minute initial perturbations. This is a defining feature of deterministic chaos. Episodes of chaotic vibrations are short lived and generally do not last longer than 10 s [43,44]. During these episodes, there is a loss of coherence in the boundary layer and a temporary stratification of fluid. The effects of these events can be seen at a distance of hundreds of micrometers. Even chaotic behavior at the submicron scale can affect hydrodynamics over large distances [45].

## 3. Multiscale Pathophysiology: From Nanoflap Oscillations to Venous Instability and Impaired Brain Clearance

### 3.1. Boundary-Layer Perturbation, Shear-Field Bifurcation, and Early Siphon Destabilization

Oscillation of the endothelial nanoflaps or nanoflap-like structures causes local perturbation of the wall region, because they are very small compared to the radius of the veins, as well as to other blood vessel sizes. Therefore, relatively small displacements (~20–30 nm) of the nanoflapse due to the large aspect ratio between the size of the nanoflap and the venous diameter will cause considerable changes in the velocity gradient close to the endothelium. Experimental and computational studies of near-wall flow dynamics using methods such as high frame rate intravascular velocimetry and PIV analysis showed that displacement amplitudes greater than approximately 25 nm would lead to alterations in the laminar velocity profiles that could be measured [46,47]. The resulting alteration in the velocity profile leads to shear field bifurcation close to the wall, where the shear vector becomes split into a strong forward directed shear vector and a weak backward directed microflow. This phenomenon has been observed experimentally; although the maximum values of shear divergence (approximately 0.1–0.3 Pa/s) appear to be quite small, it appears in regions that have very low pressure gradients (<1 mmHg). The sensitivity of the system to such perturbations means that even small disruptions to the continuity of pressure transmission along the length of the vein can occur [48].

Due to shear field bifurcation, there will also be formation of vortical structures in the boundary layer. Both the shape and duration of these vortices depend on the type of oscillation present. If the deformations are torsionally dominated then there will be elongated vortices (aspect ratio ~1.3–1.5); if the deformations are symmetrical then there will be nearly circular vortices. Further study has shown that elliptical vortices remain coherent longer than round vortices, with lifetimes of approximately 30–50 ms. The effect of these long-lived vortices extends to the downstream segments of the vein [49].

Further studies based upon pressure-field reconstructions show that each vortex generates a retrograde pressure wave having amplitudes ranging from ~0.05 to 0.12 mm Hg at its origin. Also, it has been detected that each wave propagates in the retrograde direction through distances of ~300–400 um. It has been reported that under certain circumstances, when these pressure waves overlap with those generated during peak oscillatory amplitude, transient siphon reversals occur. Siphon reversals consist of short periods (generally less than 20–30 ms) of retrograde flow rates (approximately 80–150 um/s) [50]. Even though each single siphon reversal results in only a small diminution in total volume flow, synchronization between peak oscillatory deformation and vortex generation can result in a total diminution in forward volumetric transport per drainage cycle of 4–8%. These amounts may be physiologically important, especially in areas where compensatory mechanisms are reduced [51].

In summary, all evidence supports the hypothesis that nanometer-scale oscillatory movements of endothelial cells may initiate early forms of functional instability in veins without apparent damage to structural components and potentially undetectable by current techniques of imaging [52].

### 3.2. Disruption of Perivascular Clearance: Phase Decoupling, Solute Retention Microdomains, and Astrocytic Adaptation

Hemodynamic effects occurring at the lumen surface are not isolated to the immediate vicinity of the vessel wall; they instead travel into the surrounding perivascular space, where fluid exchange depends upon coordinated pressure differences. Under normal physiological conditions, pressure differences between the venous and interstitial spaces are extremely stable (~0.1–0.3 mmHg). Therefore, even small amounts of variability in the pressure difference between the venous system and the interstitium created through oscillatory instability will create an asynchronous relationship between cardiac driven cerebrospinal fluid (CSF) pulsatile flow and interstitial fluid convective flow [53]. At baseline, under normal conditions, perivascular flow synchronizes with arterial pulsatility. However, imaging and computational studies have shown that in areas affected by oscillatory disturbances, there is an increase in the phase delay in perivenous flow relative to arterial input in some cases up to 40 degrees [54]. This loss in phase alignment demonstrates an incomplete decoupling of the perivascular pump from the overall vascular pulsatility resulting in diminished efficiency of convective transport [55].

The loss of coordination between perivascular flow and systemic arterial pulsatility results in the creation of localized retention zones of solutes referred to herein as solute trapping corridors. Solute trapping corridors are typically found along lengths of about 50–150 microns [56]. They result in lower flow velocities than their adjacent counterparts and provide solutes extended time periods for circulation. Studies employing in vivo imaging using two photon microscopy to track solute movement, DCE-MRI to measure blood flow velocity and solute concentration and fluorescent kinetic analysis have demonstrated that solute residence times are significantly greater in solute trapping corridor sections when compared to their neighboring sections. Specifically, it has been reported that tracer wash out half lives measured within the retention zone are 2.5–4 fold higher than corresponding values measured in adjacent sections [57]. Of particular note, while local rates of clearance may be maintained globally, the heterogeneity of solute transport among regions increases. Consequently, regions with solute trapping corridors tend to have elevated levels of localized gradient concentrations. Moreover, these solute trapping corridors selectively retain larger sized solutes (approximating molecular weights of 3–50 kDa), which may promote the generation of localized microenvironmental metabolic stress [58].

Additionally, rapid structural adjustments in perivascular astrocytes occur as a response to mechanical perturbations caused by oscillations. Transient cytoskeletal remodeling occurs in the astrocytic end feet located near unstable venous segments. Specifically, the cytoskeleton undergoes expansion of approximately 5–8% in surface area and reduction in cortical actin density within 100–250 ms post episode of increased oscillatory activity [59]. These structural modifications coincide with temporal and spatial rearrangements in the organization of aquaporin-4 (AQP4). Notably, temporary decreases in perivascular AQP4 density and longer term reductions in polarization indices (approximately ~5–10%) occur [60]. As previously discussed, AQP4 plays a pivotal role in mediating fluid exchange via glymphatic pathways. Therefore, even minor deviations from optimal AQP4 localization could potentially impede glymphatic fluid exchange and facilitate localized accumulation of metabolic products [61].

Taken collectively, these observations demonstrate that nanoscale oscillatory perturbations can propagate across both fluidic and cellular compartments. Furthermore, these observations highlight how such nanoscale oscillatory disturbances can lead to impaired synchronization of pressures and decreased efficiency of perivascular transport [62,63]. Table 1 represents a synthesis of how nanosecond-scale venous leaflet oscillations propagate through successively larger physiological domains, demonstrating a mechanistic continuum connecting boundary layer perturbation to impaired glymphatic transport, ionic drift in deep white matter and ultimately network level desynchronization.

### 3.3. Propagation of Oscillatory Instability into Metabolic Stress, White Matter Vulnerability, and Network-Level Consequences

Mechanical and fluid-dynamic disturbances caused by the nanoflaps oscillations ultimately imprint themselves on the surrounding tissues through successive microstress exposures. Finite element models of venous wall mechanics show that the circumferential stress concentrations at the site of chaotic oscillation events are elevated locally by 5–7%, and extend radially ~6–12 microns into the adjacent basement membrane [72]. There is a correlation between the elevation of microstress and transient disruptions of endothelial junctional continuity, therefore creating nanoscale paracellular discontinuities that allow for some extravasation of solutes. Plasma derived ions and molecules that enter the perivascular space during these episodes create local perturbations in ionic balance, thus elevating the extracellular potassium by ~0.2–0.5 mM above the resting value [73].

Even slight deviations in ionic balances have substantial effects on the electrophysiological stability of axons positioned in deep white matter tracts. Studies of conduction in these tracts show that exposure to variable extracellular ion concentrations slows action potential propagation by 0.4–0.8 ms. Such slowing is below threshold for inducing overt conduction failure; however, it can weaken the temporal coordination of conduction between fiber bundles, especially in regions of high metabolic demand and narrow conduction velocity margins [74].

Eventually, the microphysiological disturbances lead to subtle reductions in the coherence of networks. Electrophysiology-based mapping studies demonstrate that cortical and subcortical regions downstream from areas of venous instability display diminished synchrony across slow frequency bands (2–6 Hz); the degree of coherence loss varies substantially, but is typically in the range of 4–9%. The loss of coherence is correlated with the degree of impaired solute clearance and the accumulation of metabolites in the vicinity. Moreover, network modeling studies show that even small changes in the timing of conduction can result in the gradual development of drifts in oscillatory coupling, affecting cognition and adaptability [75].

Finally, impaired solute clearance can lead to localized states of oxidative and metabolic stress. Regions of the brain that have experienced chronic venous instability display increases in local oxidative marker intensity, and the concentration of reactive oxygen species (ROS) in the perivascular spaces increases by 5–12%. Oxidative stress does not uniformly affect all areas of the brain, but instead produces mosaic patterns of stress that follow the branching anatomy of the unstable venous vessels. Ultimately, such mosaic patterns of oxidative stress may contribute to the selective vulnerability of deep white matter tracts [76,77].

## 4. Molecular Mechanics of Nanoflap Resonance: The Endothelial Oscillatory Apparatus

### 4.1. Cytoskeletal Hinge Mechanics: Spectrin–Actin Assemblies and Nanoscale Rotational Compliance

The hinge area of the venous nanoflap is potentially viewed as a mechanically specialized membrane–cytoskeleton interface where force is transmitted, and deformation is synchronized through organized cortical structures. Cryo-tomographic images of very high resolution, combined with knowledge of membrane systems, imply there exist repeating actin-spectrin junctional assemblies along the hinge axis, spaced approximately every 20–25 nm [78]. While direct evidence of spectrin based lattices in live cells of venous endothelium exists, lattices formed similarly in erythrocytes, and mechanically stressed cellular membranes provide sufficient evidence that spectrin-based lattices can form along the axis of hinges in venous nanoflaps [79]. In terms of structure, each junction is a mechanical linkage site that enables controlled rotation in response to shear forces. The basis for this type of mechanical linkages involves partially extended repeat units of beta II spectrin with interconnecting actin filaments (approximately 25–35° angle relative to the plane of the membrane) connected to the plasma membrane via ERM family proteins (ezrin, radixin, moesin), thus creating a relationship between stability and mechanical adaptability, typical of membranes subjected to repeated dynamic loads [80]. Studies using both experimental and modeling approaches on spectrin-actin systems have shown that such assemblies can demonstrate rotational compliance in the range of 10^−3^ N·m^−1^·rad^−1^, therefore enabling some degree of controlled angular movement in response to applied forces [81]. Additionally, it is known that spectrin repeats can undergo reversible axial extension (~5–10%) that can store elastic potential energy temporarily during deformation cycles [82,83]. Time constants for the relaxation of extensions were found to be in the submillisecond range (~0.8–1.3 ms), indicating that the cytoskeletal network can respond on timescales that match those required to generate high frequency oscillatory motion [84].

Tension-dependent regulation of cytoskeletal protein interactions will also modify the mechanical characteristics of the hinge. For example, proteins containing calponin homology domains (such as α-actinin related proteins) may regulate the binding affinity between actin and spectrin in a manner dependent upon intracellular tension [85]. Therefore, under increased mechanical load such mechanisms would result in decreased compliance (estimated ~8–12%) at specific points along the hinge axis, reducing excessive rotational displacement. However, unlike previously envisioned “structural nodes”, these effects should be considered as distributed, tension sensitive modifications in cytoskeletal stiffness, consistent with established models of cortical mechanoadaptation [86].

Therefore, the data supports the concept that the hinge functions as a dynamically regulated mechanical interface that integrates shear forces and regulates rotational compliance. Although the detailed molecular architecture of venous nanoflaps has not been directly visualized in living tissue, the proposed behavior is consistent with all known properties of membrane-cytoskeleton systems [87]. Consistent with this perspective, variations in cytoskeletal organization (due to genetic factors, metabolic states, or post translational modifications) may plausibly affect threshold levels for oscillation and mechanical sensitivity and subsequently impact local flow-structure interactions [88].

### 4.2. Membrane Viscoelasticity and Lipid-Lattice Architecture: Curvature-Distribution Networks, Shear-Damping Corridors, and Anisotropic Flexural Fields

The lamellar body of the venous nanoflap can be thought of as a mechanically heterogeneous membrane system. The viscoelastic properties of the lamellar body arise from the spatial organization of lipid phases and their interaction with membrane associated proteins. Studies utilizing superresolution microscopy techniques and membrane biophysics have provided evidence that there exist ordered lipid micro-domains rich in sphingomyelin and cholesterol that form semi-regular arrangements with average dimensions approximately 20–30 nm [89,90].

Ordered lipid domains are typically stiffer than disordered lipid domains due to tighter packing order and reduced lateral diffusion rates. Given its heterogeneous organization, the lamellar body exhibits anisotropic flexural behavior where resistance to bending depends on the directionality and orientation of applied forces. Specifically, because ordered lipid domains are resistant to deformation when bent along their alignment axes, adjacent regions with higher concentrations of unsaturated lipids allow greater curvature adaptation [91]. This type of anisotropy is well documented for biological membranes and provides a rationale for why direction dependency exists for mechanical responses during oscillatory deformation. Adjacent to ordered lipid domains are more fluid lipid environments rich in polyunsaturated phospholipids. Polyunsaturated phospholipids have lower packing densities compared to ordered lipid domains and thus exhibit higher deformability. Regions rich in polyunsaturated phospholipids have reported viscosities ranging between approximately 0.3–0.6 Pa·s [92].

Given these physical properties, fluid lipid regions are likely responsible for absorbing energy generated during oscillatory deformation. Fluid lipid regions dissipate energy generated by oscillatory deformation as opposed to storing excessive strain. Finally, additional mechanical modulation may occur due to curvature sensitive lipid assemblies. Curvature sensitive lipid assemblies include lipid domains enriched with lipids having inherent curvature (i.e., lysophospholipids) [93,94]. Domains enriched in curvature sensitive lipids with diameters approximately equal to 10–20 nm can locally adjust curvature fields and facilitate redistributions of bending stresses. Using computational methods researchers estimated that curvature sensitive lipid assemblies could account for a reduction in variance in bending energies during torsional deformation by approximately 10–20% [95].

**Figure 2:**
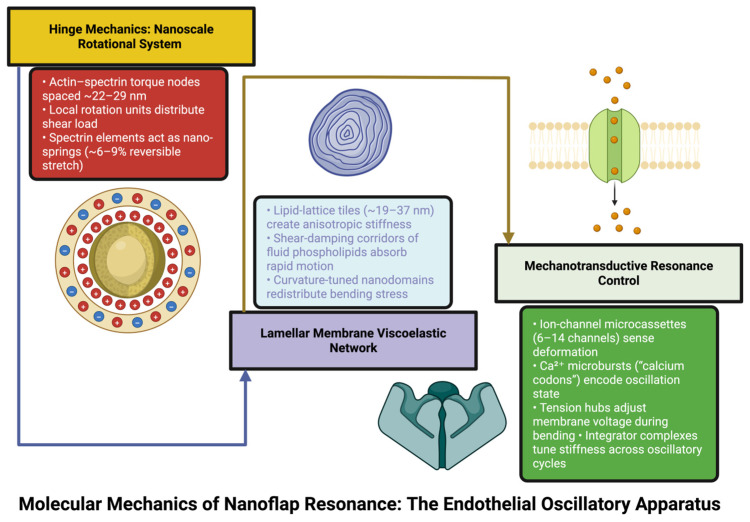
Proposed molecular interaction network supporting venous nanoflap resonance behavior. The schematic highlights the coupling between three regulatory layers: a cytoskeletal hinge module, in which spectrin–actin assemblies provide force distribution and reversible rotational compliance; a lamellar membrane module, in which lipid heterogeneity shapes local stiffness, damping, and stress redistribution; and a mechanotransductive module, in which mechanosensitive ion channels and localized Ca^2+^ signaling provide activity-dependent feedback to membrane and cytoskeletal mechanics. The figure is intended to illustrate how these molecular subsystems may interact to stabilize, amplify, or modulate oscillatory states under changing hemodynamic conditions.

Estimates made using this approach are model derived and correspond to known principles governing curvature elasticity. Collectively, these observations provide a model in which the lamellar membrane is composed of multiple components that function cooperatively to create a viscoelastic structure. Specifically, ordered lipid domains are responsible for providing mechanical support to the lamellar membrane; fluid regions are responsible for dissipating oscillatory energy; and curvature sensitive lipid assemblies facilitate redistributing bending stresses [96]. This cooperative organization enables nanoflaps to maintain structural integrity while accommodating dynamic mechanical loading. Notably, this perspective highlights that oscillatory behavior arises from cooperative interactions between cytoskeletal mechanics and membrane viscoelasticity and not from a single structural element [97]. Figure 2 illustrates these cooperating subsystems–cytoskeletal hinge dynamics, membrane mechanical heterogeneities and associated mechanotransducing elements—within a comprehensive, although hypothesis driven, framework of venous nanoflap resonance.

### 4.3. Mechanotransductive Resonance Control: Ion-Channel Microdomains, Localized Calcium Signaling, and Multi-Timescale Regulatory Integration

Mechanical forces exerted on the nanoflaps oscillations do not solely depend on mechanical properties; they also rely upon closely linked mechanical transduction systems. The mechanically induced deformation at the junction of the hinge and lamellae is converted into coherent biochemical reactions through mechanotransduction. Mechanosensitive ion channels are found to be organized in a defined manner, rather than being randomly distributed. In fact, these have been named “ion channel microcassettes” [98,99]. The dimensions of these microdomains vary between 70–120 nm. Each contains about 6–14 mechanosensitive ion channels. Some examples include the well-documented endothelial mechanoreceptors PIEZO1, TRPV4 and ASIC family members. In addition, each microdomain has supporting local scaffolding elements that help to coordinate channel activity when subjected to shear-induced deformation [100].

Localized calcium signaling is generated from oscillating mechanical stresses acting on these microdomains. Thus, it can be said that the mechanically stimulated calcium signaling is “spatiotemporal” or “temporally/spatially limited.” Each event has a duration of about 5–12 ms [101]. Furthermore, calcium signaling remains localized for distances up to 200–400 nm. Therefore, it appears that this type of calcium signaling is a structured and repeatable response to mechanical stimulation. As such, the time/space characteristics of Ca^2+^ signaling represent a ‘signature’. It is known that the time/space characteristics of Ca^2+^ signaling determines downstream functions in mechanosensitive cells [102].

These calcium microtransients trigger rapid and spatially-localized cytoskeletal remodeling events, i.e., actin reorganization, and adjustments of ERM-mediated membrane-cytoskeleton interactions. Calcium signaling acts as an active feedback regulator to allow the nanoflap to adjust its hinge flexibility/stiffness and lamellar compliance continually in accordance with changing hemodynamic forces. In addition to calcium-mediated signaling pathways, there are several other regulatory pathways present in the form of additional regulatory components [103,104]. Specifically, voltage-dependent and stretch-sensing membrane complexes were termed as “ionotropic tension hubs.” Ionotropic tension hubs integrate mechanical strain and ionic fluxes to modulate local membrane potentials, with estimated variations of about 1–2 mV during cyclic deformations. Activation thresholds of adjacent ion channels and their downstream effectors are regulated by ionotropic tension hubs. This enables them to tune cytoskeletal tension and oscillatory sensitivity across the lamellar surface [105].

There are also slow adaptative mechanisms operating at longer timescales in nanoflap regulation. Oscillatory-state integrator complexes, likely containing phosphatidylinositol (PI)-annexins and PI(4,5)P2-rich phospholipid domains, respond not to instantaneous deformation but to the total accumulated mechanical load applied to the system. Prolonged oscillatory loading elicits localized remodeling of membrane lipid composition with small but physiologically significant increase in membrane stiffness (~6–10%) due to their slow kinetics (>100 ms). They thus function as stabilizers and prevent gradual shifts in oscillatory behavior as well as reduce susceptibility to chaotic state transitions resulting from varying flow conditions [106].

**Figure 3:**
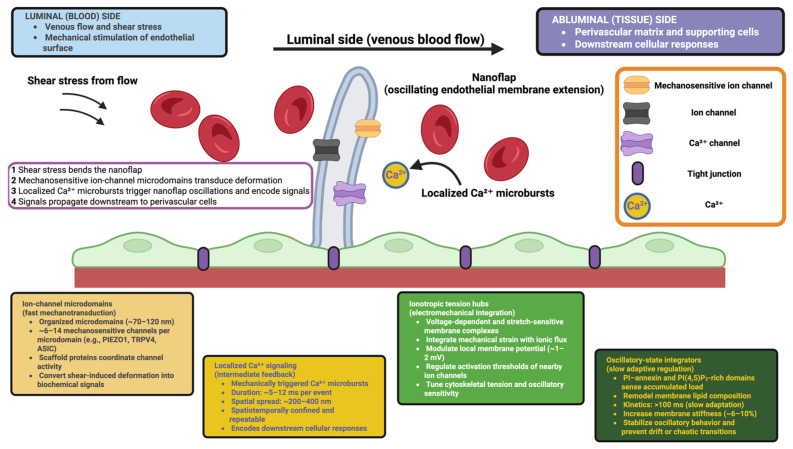
Mechanotransductive regulation of venous nanoflap oscillations. Conceptual schematic of a proposed venous endothelial mechanotransduction system in which luminal shear stress deforms endothelial membrane extensions (“nanoflaps”), activating mechanosensitive ion-channel microdomains, localized Ca^2+^ microbursts, and downstream endothelial–perivascular signaling. Fast, intermediate, and slow regulatory modules coordinate oscillatory dynamics, membrane adaptation, and mechanobiological stability.

Collectively, the above mechanisms establish a hierarchical organization of a mechanotransduction pathway. Rapid ion channel responses, intermediate calcium mediated feedback and slow lipid-protein based adaptation provide multiple levels of regulation controlling nanoflap resonance. Such multi-level control structure provides oscillatory stability while enabling responsiveness to both short-term acute mechanical disturbances as well as long-term changes occurring in the surrounding endothelial environment [107]. To facilitate interpretation of these coupled processes, Figure 3 schematically integrates the principal regulatory levels proposed to govern venous nanoflap mechanotransduction within a single multiscale framework.

## 5. Conceptual Approaches to Modulating Venous Nanoflap Mechanics: From Molecular Targets to Hypothesis-Driven Therapeutic Directions

### 5.1. Hinge-Directed Modulation: Targeting Cytoskeletal Compliance and Spectrin–Actin Dynamics

Within the proposed framework, the mechanically significant interface is represented by the hinge region; here, the cytoskeletal organization is responsible for translating shear stress into rotational motion. Therefore, the modulation of hinge mechanics is a possible approach (although still theoretical) to modulating the oscillatory behavior of nanoflaps. It is important to note that the goal of such modulation will not be to fixate the hinges rigidly, but to fine tune their torsional compliance to a regime that is physiologically stable [108].

At the molecular level, it is expected that the hinge is primarily composed of βII-spectrin–actin junctional complexes anchored to the membrane via ERM. Spectrin repeats have been characterized as undergoing well-defined force dependent conformational transitions (reversible unfolding and axial elongation) up to ~5–10% strain. The fact that these repeat structures can store and release elastic energy indicates that spectrin functions as a molecular spring [109]. From a mechanistic perspective, one possible way to modulate the hinge mechanics would be to modify the flexibility of the spectrin repeat units. Examples of modifications include altering the interactions of spectrin with binding sites between adjacent repeats or modifying the stability of α–β spectrin interfaces. Such alterations in theoretical terms could lead to a decrease in the degree of elongation upon force induced conformational transition; consequently, potentially preventing excessive elongation during high frequency oscillatory cycles. Computational models predict that moderate (~10–15%) increases in effective torsional stiffness could move the system out of non-linear or unstable oscillatory regimes. However, since these predictions have yet to be tested in endothelial cells, caution must be exercised [110].

A complementary method for modulating the mechanics of the hinge would be to modulate its hysteretic behavior, i.e., the energy lost due to repeated unfolding/re-folding of spectrin. Force spectroscopy of single molecules has shown variation among different repeats with respect to their elongation profiles; thus, decreasing this variation (e.g., changing elongation ranges from ~6–9% to ~3–5%) could generate smooth force—extension curves and reduce oscillatory irregularities. It is also important to note that such effects must maintain the inherent frequency response of the hinge so as to prevent disruption of physiological oscillatory coupling [111,112].

Furthermore, actin cross-linking dynamics that are influenced by α-actinin and similar proteins could also affect how torsionally generated energy is transferred across the hinge. Tension-dependent alterations in protein-protein interactions can influence local stiffness gradients and possibly diminish the likelihood of localized over-deformations. Instead of viewing these processes as “gatekeeper” structures, we view them as continuous and load-dependent adjustments in cytoskeletal coupling efficiencies, which are consistent with previously established models of cortical mechanoadaptation [113].

Taken collectively, our understanding suggests that the hinge could be viewed as a tunable mechanical system. Small adjustments at the molecular scale may be able to influence oscillatory thresholds and stability. Nevertheless, all of these possibilities remain speculative until they are experimentally verified.

### 5.2. Lamellar Membrane Modulation: Lipid Phase Behavior and Viscoelastic Regulation

Another potential axis for theoretical modulation of oscillatory behavior lies in lipid-mediated modification of bending stiffness, viscosity and dissipated energy of the lamellar component of the venous nanoflap [114,115].

One structural aspect is the existence of liquid ordered micro-domains rich in sphingomyelin and cholesterol with lateral dimensions of approximately ~20–30 nm. Liquid ordered domains show increased ordering of lipids and decreased lateral mobility compared to disordered membranes, resulting in increased stiffness and less susceptibility to deformation. Studies have indicated that variations in lipid packing order parameters (S), on the order of ~0.05–0.1 can cause significant variations in membrane bending rigidity. Thus, theoretically, modulation of lipid packing—e.g., through changes in cholesterol distribution or sphingolipid interactions—could increase structural cohesion and reduce anisotropic deformation under oscillatory stress [116].

Complementary to these ordered domains exist more fluid membrane areas containing polyunsaturated phospholipids that show lower packing densities and greater deformability. The effective viscosity of these membrane areas is ~0.3–0.6 Pa·s, allowing efficient dissipation of mechanical energy during high frequency deformation. Theoretically, modification of these properties—through variation in acyl chain flexibility or lipid–protein interactions—could affect shear damping capability, potentially limiting propagating mechanical stress waves and reducing oscillatory overshoot [117].

An additional mechanism for controlling membrane mechanics could result from curvature sensitive lipid assemblies such as domains rich in lyso-phospholipids or phosphatidylethanolamine species. These lipids inherently exhibit curvature preference and can modulate local membrane geometry. Model simulations indicate that redistribution of such domains could decrease spatial heterogeneity in bending energy by ~10–20%, thereby improving stability of oscillatory waveforms during torsional loading. Although these values were obtained using a computational model and do reflect known principles of membrane curvature elasticity, they are entirely predictive and require experimental verification [118,119].

Together, the lamellar membrane may be thought of as a multi-modal mechanical system where mechanical stiffness, plasticity, and energy loss are dynamically coupled through lipid organization. Although there are technical challenges to pharmacologically manipulating these properties, the above represents a theoretical framework for regulating oscillatory behavior at a membrane level that has yet to be experimentally investigated [120].

### 5.3. Mechanotransduction-Based Modulation: Ion Channel Dynamics and Calcium Signaling

Finally, another layer of potential modulation exists with regard to mechanotransduction-based signaling pathways that convert mechanical deformation into biochemical responses. Since Piezo1 and TrpV4, two primary mechanosensory ion channels found in endothelial cells, play major roles in mechanosensation, modulation of these channels could regulate oscillatory dynamics via negative feedback mechanisms on cytoskeletal tension and membrane properties [121].

Mechanosensitive channels have activation thresholds dependent on membrane tension, curvature and lipid composition. Experimental evidence shows that relatively small variations in activation threshold (typically ~5–10%) can greatly impact channel responsiveness. Conceptually speaking, regulation of these activation thresholds could minimize spontaneous or noise driven channel opening events thereby providing stability for oscillatory cycles and minimizing amplifications due to minor disturbances [122].

Down stream of channel activation, localized Ca^2+^ micro-transients function as key regulators in mediating cytoskeletal responses. Typically, Ca^2+^ micro-transients last ~5–12 ms and propagate ~200–400 nm radially within endothelial cells [123]. Modulation of Ca^2+^ signaling (channel kinetics, buffering etc.) or regulation of Ca^2+^ micro-domain formation could influence both the temporal resolution and spatial localization of mechanochemical signaling. For instance reduction in time course or radial extent of Ca^2+^ transients could reduce excessive contractile activity and allow improved local control over mechanical responses [124]. Longer timescale regulation may occur through signaling pathways regulated by phosphoinositides especially PI(4,5)P2 which play a key role in coupling membranes with cytoskeletons and regulating channel activities. Stabilization of phosphoinositide nano-domains could help stabilize drifts in membrane stiffness and cytoskeletal tension over multiple oscillatory cycles. Models suggest that such mechanisms could reduce long term variability in mechanical properties by ~30–40%. However, since experimental validation is required to confirm this prediction in this context caution must be used [125].

Collectively, these mechanisms demonstrate that mechanotransductive signaling pathways represent a dynamic regulatory layer that links mechanical deformation with adaptive cellular responses. Similar to other strategies presented herein; these ideas remain hypothesis-driven and must be validated in physiological contexts [126].

### 5.4. Future Directions: Toward Integrated and Adaptive Modulation of Oscillatory Systems

Possible future directions for developing new strategies for modulating oscillatory systems may lie in developing multiscale and adaptive modulation strategies that integrate structural membrane and signaling mechanisms. Rather than individually target specific components, these approaches would attempt to adjust the overall energy landscape governing oscillatory behavior while maintaining stability throughout physiological frequencies (~40–110 Hz) while avoiding entry into non-linear/chaotic regimes [127]. In theoretical models describing oscillatory systems, the relationship between torsional mode bending modes is generally described by dimensionless interaction coefficient estimates (~0.4–0.6) under normal conditions. Modifying these relationship coefficients could potentially reduce higher order harmonic generation and/or stabilize waveform structure; however, these types of approaches remain purely conceptual at this point [128].

Another emerging direction will be developing adaptive mechanobiology based modulation strategies that adaptively respond to local hemodynamic conditions. Thus theoretically small context-specific adjustment in mechanical properties (approximately ~2–4%) could serve as buffer to transient fluctuations in shear forces thereby maintaining stability in oscillations without compromising physiological responsiveness [129]. Table 2 organizes developing pharmacologic strategies for stabilizing venous nanoflap dynamics by affecting their fundamental nanomechanical determinants.

## 6. Translational Implications: Oscillatory Venous Nanomechanics Across Neurological Disease States

### 6.1. Neurodegenerative Contexts: Oscillatory Drift, Metabolite Retention Microenvironments, and Axoglial Vulnerability

Altered vascular-metabolic coupling and impaired clearance mechanisms have been identified as a feature of neurodegenerative conditions. From this perspective, the oscillatory nanoflap model provides a basis for understanding how subtle alterations in oscillatory behavior may contribute to unstable micro-environmental conditions; however, the nature of the relationship between these two factors remains undefined [142,143]. Indirect modeling data and experimental evidence suggest that oscillating systems subject to altered biochemical conditions may exhibit slow drift in their resonance frequencies over time. The magnitude of this drift would vary depending upon the duration and intensity of exposure, and may be approximately equal to 0.3–0.7 Hz/h during prolonged exposures [144]. Given that these drifts represent gradual changes in mechanical and biochemical properties at the local scale, including oxidative stress, membrane composition and cytoskeleton regulation, it is possible that such changes could contribute to decreased oscillatory coherence. Such a decrease in coherence could result in localized accumulations of partially degraded oxidative products including lipid peroxides and small aldehydes. Importantly, these “micro-domains” need not be indicative of primary metabolic dysfunction, but rather represent inefficient synchrony in clearance between the venous compartment and the interstitial space [145,146].

This represents a different concept than previously described solute retention phenomena and needs further experimental validation. It is reasonable to hypothesize that similar micro-environmental changes could affect mechanical interactions between axons and glia, particularly near perivascular regions. Although the nature of these changes is currently unclear, transient alterations in the mechanical properties of myelin in response to low amplitude mechanical perturbations (“axo-glial vulnerability”) may occur [147]. Models of myelin dynamics indicate that small changes in parasomal stiffness (e.g., approximately 3–5%) may significantly affect sodium channel cluster formation and function under stressful conditions. Instead of resulting in persistent delays in nerve impulse conduction velocity, such effects may cause intermittent variability in conduction velocity. Intermittent variability in conduction velocity has been shown to precede network desynchronization in some experimental systems [148].

Similarly, disruptions in oscillatory coupling may affect lipid metabolism within the neurovascular unit. Specifically, disruptions in coordination between astrocyte-mediated lipid efflux and vascular clearance may lead to temporal mismatches between lipid release and clearance [149]. Temporal mismatches between lipid release and clearance may lead to transient perivascular accumulation of lipids and increased metabolic burden on surrounding tissues [150]. As an example, if lipid transport processes were synchronized with slower vascular oscillations under normal conditions, disruptions in this synchronization might lead to temporal mismatches between lipid efflux from astrocytes and clearance from veins. These mismatches might lead to transient perivascular lipid accumulation and subsequent increased metabolic burdens on surrounding tissues. Although this concept—termed “lipid turnover desynchronization”—is speculative at this point, it is supported by emerging research suggesting that lipid metabolism is disturbed in many neurodegenerative diseases [151].

Collectively, these concepts support the hypothesis that oscillatory disturbances occurring at very fine scales (nanometer-length scales) may serve as early indicators of neurodegenerative disease susceptibility. Moreover, these disturbances may act to reduce clearance efficiency and/or destabilize micro-environments; however, these hypotheses are based solely on speculation and require experimental validation [152].

### 6.2. Disorders of CSF–Venous Compliance: Intravascular Compliance-Phase Shifts, Distortion of Low-Frequency Pressure Harmonics, and Compliance-Memory Loss

Diseases characterized by abnormal CSF-venous interactions (communicating hydrocephalus, idiopathic intracranial hypertension, etc.) include abnormal CSF-venous pressure-flow couplings. Similarly, abnormal nanoscale oscillatory systems—specifically those involved in low-frequency compliance dynamics—are likely affected by such disorders [60]. Physiological conditions demonstrate coordinated behavior between venous and CSF systems within low-frequency harmonic bands (approximately 0.1–0.3 Hz). Therefore, it is logical that nanoscale endothelial structures contribute to this coordinated response through phase-alternated mechanical adaptations. Pathological conditions, conversely, likely disrupt this coordination causing phase-lag between mechanical response and pressure stimulus [153].

Theoretical modeling indicates that mechanical response lags on the order of 15–30 ms will modify the amplification of low-frequency harmonic components thereby modifying overall compliance characteristics of the intracranial system. Since these values are theoretically-derived from indirect measurements and previous research regarding coupled oscillatory systems sensitivity to phase-lag, they provide a useful conceptual framework [154].

Additionally, loss of adaptive compliance behaviors (often referred to as “mechanical memories”) may occur due to disruption of cytoskeletal remodeling and mechanotransductive signaling processes. Loss of these adaptive behaviors likely results in variable compliance from one cycle to another (as opposed to cumulative adaptation) leading to increasingly disparate local stiffness values thereby reducing capacity for intracranial pressure buffering. Mechanisms underlying loss of compliance memory are likely involved with phosphoinositide signaling, cytoskeletal turnover and ion-channel regulation rather than actual memory storage [155].

At the tissue level, such loss of coordinated compliance behavior likely contributes to heterogenous compliance throughout the periventricular region(s). Heterogeneous compliance within this region(s) likely affects local pressure loads activating mechanosensitive pathways within astrocytes and other glial cell populations. Notably, these effects are consistent with known principles of neurovascular coupling; however, the linkages to nanoflap dynamics remain speculative and require empirical evaluation [156]. Therefore, the proposed framework suggests that disorders involving impaired CSF-venous compliance behave as multi-scale regulatory failures (i.e., failure of coordinated regulation across scales ranging from systemic dynamics to individual endothelial cell mechanics) in addition to being recognized as macroscopic regulatory failures [157].

### 6.3. Pulsatility Disorders, Post-Infectious Dysautonomia, TBI Spectrum, and Microcirculatory Stress

Post-infectious dysautonomia, traumatic brain injuries (TBIs), and systemic micro-circulatory stress disorder share common features including alterations in pulsatile signaling/vascular regulation and therefore potentially disrupting entrainment between systemic rhythms and local endothelial oscillatory dynamics [158]. Under normal physiological conditions, venous nanoflaps exhibiting oscillatory behavior would be expected to exhibit entrainment to systemic pulsatile activity including autonomically regulated vasomotor rhythms. Conversely, irregularities in vasomotor rhythms within the same frequency band (approximately 0.05–0.15 Hz) likely create non-uniform shear stresses affecting local mechanical responses to vasomotor stimuli and therefore disrupt entrainment between systemic inputs and local mechanical responses [159,160].

This disruption may be viewed as a loss of oscillatory coherence between systemic rhythm inputs and local mechanical responses creating increased variability in oscillatory patterns. Trauma-induced mechanical disruption of endothelial/perivasculature structures may temporarily alter spatial coherence of oscillatory behavior [161]. Rather than representing physical destruction of structures (i.e., structural damage), functional desynchronization between cytoskeletal/membrane dynamics within each nanoflap structure is likely responsible for irregular propagation of mechanical signals through the structure. Conceptually referred herein as “oscillatory fragmentation,” this represents a hypothetical description of functional disruption as opposed to structural disruption [162].

Disruptions caused by systemic hypoperfusion or micro-circulatory shock likely induce new relationships between shear-stress levels and oscillatory behavior. In theory, this alteration may lead to decoupling between driving frequency and local oscillatory response thereby inducing non-linear stress distributions within cytoskeletal networks. Described herein using terminology analogous to “resonance inversion,” it represents a conceptualization of loss-of-proportional coupling observed in driven oscillatory systems; however, it does not refer to an empirically-measured biological process. Together, these examples demonstrate how venous nanoflap dynamics (if present) may act as integrators of systemic perturbations transforming alterations in systemic pulsatility/mechanical inputs into local endothelial/microvascular responses. However, it is crucial to emphasize that all hypotheses presented are speculative in nature and require experimental validation [163,164].

## 7. Future Directions: Predictive Venous Nanomechanics, Resonance Cartography, and Multiscale Neurovascular Intelligence

### 7.1. Ultra-Future Imaging Platforms: Quantum-Sensitivity Resonance Scopes, Intraluminal Nanoflap Interferography, and Oscillatory Biome Datasets

The future of venous nanomechanical research depends on its ability to visualize submicron-scale endothelial oscillation under physiological conditions. Current standard imaging modalities do not permit visualization of the very small scales of endothelial cell movement. There are however new technologies developing that can enhance our ability to see submicron scale movements of endothelium. Nitrogen-vacancy (NV) center in diamond quantum enhanced sensing has been used for observing nanoscale movements of lipid bilayers and cytoskeleton. Theoretically an NV center sensor attached to a biological interface could indirectly measure time-dependent oscillatory patterns caused by membrane deformation and/or cytoskeleton movement; however many challenges still exist for NV center technology to be able to attach to biological surfaces, target specific areas and interpret the data [165,166].

Optical interferometry and other forms of optical phase-sensitive imaging have also developed and may offer alternative options. Phase-sensitive interferometry, especially using fiber optic or waveguide technology may allow us to observe nanometer displacement with millisecond resolution. As we continue to improve these technologies, they may provide us with the capability to measure the motion of the endothelial surface and capture both the amplitude and phase information about the motion, as well as study the spatial distribution of oscillatory waves and identify if this type of motion is confined to limited areas of the vein or propagated throughout the vessel [167].

Ultimately, a major challenge facing researchers in the field today is creating multidimensional and time-resolved vascular maps that integrate structural, blood flow and pressure-related information. These types of maps would allow investigators to examine common patterns of oscillatory motions, compare the frequency of oscillatory motions between individuals, and develop criteria for diagnosing diseases related to abnormal oscillatory motion. At this point in time, creation of these datasets is considered long-term objectives, requiring additional technological advancements [168,169]. Together, these technologies outline a feasible path forward for studying nanoscale vascular dynamics, but require incremental Progress toward validating nanoscale vascular dynamics

### 7.2. Computational Neurofluidics: AI-Driven Resonance Solvers, Mechanome Predictive Fields, and Multi-Harmonic Risk Architectures

Computational modeling is expected to be an essential tool in understanding the feasibility and significance of vascular nanomechanics due to limitations in experimental methodology. To achieve this, fluid dynamics, membrane mechanics, and endothelial signaling will all need to be incorporated into a unified multiscale model. High-dimensional parameter exploration models: one way to address some of the difficulties in predicting how nanoscale vibrations are influenced by parameters like membrane thickness, membrane viscosity, and channel opening thresholds is to use high-dimensional parameter exploration models [170]. If a researcher were interested in simulating how variations in these parameters affected oscillatory behavior over a wide range of conditions, he/she could use machine-learning techniques to explore large mechanical parameter spaces. Instead of making determinative predictions, these models would help researchers find the regions where their models become unstable, the transition zones between different states, and the regions of greatest sensitivity. They would therefore provide a quantitative foundation upon which researchers could test their hypotheses [171,172].

Modeling local mechanical disturbances in venous networks: a second strategy involves modeling how localized mechanical disturbances in boundary layer flow affect downstream pressure fields and perivascular transport in venous networks. Researchers can predict how far nanoscale disturbances will travel down a network and what kind of impact they will have on transport and flow using computational fluid dynamics. By determining whether or not small-scale oscillations quickly lose energy as they move down a network or if there are any conditions under which they are capable of traveling farther and influencing larger-scale flow structures, researchers can gain insight into how much effect small-scale oscillations are likely to have on overall flow stability [173].

Modeling interacting oscillatory elements along venous pathways: finally, researchers can investigate how various elements along a pathway interact to produce emergent behaviors such as synchronization, phase shift and non-linear coupling. It is well established in physics that systems containing multiple interacting oscillators will often display complex dynamic behaviors dependent on the nature of the interactions between them. Therefore applying similar ideas to systems consisting of interacting oscillatory elements along venous pathways may reveal whether or not the previously proposed nanoflap dynamics could meaningfully influence venous flow stability [174].

It is important for researchers to view computational methodologies as tools for limiting/defining hypotheses and not as replacements for experimental validation. The primary benefits provided by computational methodologies include: (a) identifying those components of proposed frameworks that are most sensitive, testable and physiologically relevant [175].

### 7.3. Integrative Neurovascular Systems: Resonance-Governed Connectomics, Biomechanical State Plasticity, and Nano-Governed Metabolic Architectures

An important step forward in understanding vascular nanomechanics will be linking it to broader contexts such as neurovascular function and neurophysiology. This entails transitioning away from isolated structural models of vascular dynamics toward more integrated analysis of flow, metabolism and neural activity [176]. Linking vascular dynamics to white matter conduction timing: an important consideration will be examining whether vascular dynamics can influence white matter conduction timing [177]. It has been observed that conduction velocity is sensitive to micro-environmental factors including ionic balance, metabolic support, and extracellular pressure. Since there is no direct evidence indicating that nanoscale oscillatory behavior affects conduction timing, it is conceivable that subtle spatially heterogeneous perturbations in perivascular conditions can influence signal transmission timing across distributed neural networks. These types of influences are likely to be small and cumulative, thus necessitating high-resolution experimental systems to assess [178,179].

Adaptive changes in vascular mechanical properties over time: another area of interest will be understanding how vascular mechanical properties undergo adaptations over time that are responsive to metabolic demands, sleep-wake cycles, inflammatory responses and aging [180]. Unlike structural remodeling, these changes will occur gradually through alterations in mechanical responsiveness that may result from turnover in cytoskeletal proteins, changes in lipid compositions and modifications of mechanotransductory signaling pathways. Investigating this type of mechanical plasticity may shed light on how vascular systems maintain homeostasis under prolonged periods of stress and how loss-of-function in this type of plasticity contributes to disease [176,181].

As well, there is growing recognition that vascular dynamics are intimately linked to local metabolic regulation including redox state, nutrient delivery, and astrocyte mediated support. Whether the proposed concept that nanoscale mechanical phenomena directly influence metabolic gradients holds true remains uncertain; nonetheless it is consistent with the broad tenet that fluid dynamics and cellular metabolism are intricately intertwined within the neurovascular unit. The evaluation of this possibility will require interdisciplinary investigations involving imaging, modeling and metabolic analyses [182,183].

Collectively these viewpoints indicate that venous nanomechanics—if experimentally verified—could serve as one component part of a multi-scale regulatory system relating endothelial structure, hemodynamics and brain function. However integration of these systems represents a conceptual entity at this stage of knowledge development and should be pursued through incremental investigations [184].

### 7.4. Conceptual Boundaries and Evidentiary Context

Several important considerations need to be addressed as limitations. More in-depth studies on the structure of venous nanoflaps (in vivo) along with the oscillating behavior of these structures exist as little more than theoretical speculation. Therefore, it would be premature to establish the venous nanoflap as an anatomical entity based upon the current data. Likewise, many quantitative values that will be discussed throughout this paper are derived through model based estimations, indirect observation and/or analog systems. These values do align with established physical principles however, they should be viewed as working hypotheses rather than established physiological parameters.

Additionally, while there appear to be reasonable links between the mechanistic activity of nano-scale endothelial function and larger scale vascular/neurological consequences; these relationships are speculative. While the proposed links align with well-established concepts regarding neurovascular coupling and fluid-structure interactions; the exact nature of these potential causal pathways has not been definitively shown. It is entirely possible that other mechanisms could explain the proposed relationships such as systemic hemodynamic factors, astrocyte regulation of capillary permeability, inflammation, etc., and/or metabolic abnormalities.

As previously stated, the majority of the work presented within this review was generated from a bottom-up approach; therefore, we have only begun to examine reciprocal effects where large scale/systemic states influence small scale/endothelial mechanical properties. Finally, it is anticipated that the ideas presented here will serve as potential avenues for further research and/or development into new treatments.

## 8. Conclusions

This study advances an interpretive framework for understanding the biomechanics of deep cerebral veins based on multiple length scales; specifically, macroscopic flow patterns are generated through interactions between pressure gradient induced flow behaviors and biomechanical events occurring at the nano-scale (i.e., at the endothelial interface). These nano-scale biomechanical events include the operation of venous “nanoflaps” as candidate oscillators to induce localized, near wall flow features which can destabilize the laminar boundary layers and cause local discontinuities in pressure continuity.

One key aspect of the studies findings was the ability to merge information from across the structural, molecular and hydrodynamic domains. Specifically, using molecular models, we were able to describe several possible candidates for mechanically driven oscillation and deformation sensitive signaling at the nano scale including: hinge associated ERM-spectrin-actin complexes; mechanically disordered lipid micro-domains; and mechanically gated ion channels such as PIEZO1 and TRPV4. We propose that these biomechanical events could cause transitory micro-scale shear distributions and vortex formations. Further, these events could produce localized micro-scale retrograde pressure pulses. Finally, the impact of these micro-scale events could lead to small scale deviations in large scale venous flow properties resulting in altered perivascular transport characteristics and potentially localized differences in solute clearance.

As important, this synthesis indicates that abnormal cerebral venous physiology may occur in the absence of grossly apparent structural defects in vessels. This may result from disruptions in oscillatory coherence and phase relationships between biomechanically driven and hydro-dynamically driven mechanisms. Furthermore, such disruptive mechanisms would likely not be detectable via conventional imaging modalities. Therefore, they would represent a previously unappreciated level of regulation in neurovascular functions. As such, this integrated model provides a plausible link between the biomechanics of the endothelium and two poorly understood phenomena; namely, impaired glymphatic transport and localized accumulation of metabolic waste products. However, it is essential to recognize that there are critical evidence gaps in this conceptualization. That is, although individual aspects of this model have been demonstrated experimentally; i.e., nanoscale membrane mechanics, endothelial mechanotransduction and near wall flow perturbation; these individual components have not yet been integrated into a single model of venous nanoflaphyamics. For example, quantitatively estimating biomechanical variables such as deformation frequency, amplitude, etc.; relating these biomechanical variables to measurable effects in venous flow and perivascular transport; establishing biomechanically relevant mechanical properties for specific molecules or proteins; etc. All of these issues require further research. However, some aspects of this research area may become tractable soon due to recent developments in high resolution imaging techniques both in vitro and in vivo. Additionally, developing computational modeling tools incorporating biologically realistic mechanical properties of cells with fluid dynamic simulations of blood flow will allow us to test whether or not our hypotheses are consistent with existing physiological conditions.

The pharmacological interventions described in this manuscript should be viewed similarly as illustrative examples of potential new ways to modify vascular function through mechanical means. They do not constitute proven therapeutic options. However, they represent a possible conceptual direction towards identifying additional mechanical factors influencing vascular function such as hinge compliancy, membrane viscoelasticity and sensitivity thresholds for mechanoreceptive signaling pathways.

## Figures and Tables

**Table 1:** Details each hierarchical tier of dysfunction—ranging from shear-field bifurcation and vortex persistence to astrocytic AQP4 depolarization, paracellular leakage, conduction delay, metabolic microstress, and coherence drift—highlighting the quantitative signatures and spatial scales characteristic of each step.

Proposed Domain	Representative Nanoscale/Local Process	Localized Consequence	Possible Higher-Order Implication	References
Near-wall flow instability	Oscillatory displacement > 25 nm, shear-field bifurcation, altered vortex geometry	Short-lived microvortices (35–55 ms), small retrograde pressure pulses (0.07–0.12 mmHg), brief flow reversal (90–150 µm/s)	Reduced efficiency of forward venous transport (~4–8%) and early siphon instability below conventional imaging thresholds	[64]
Pressure-field irregularity	Shear divergence of 0.1–0.3 Pa·s^−1^ across short venous segments	Flow segmentation, unstable velocity gradients, intermittent local backflow	Potential attenuation of venous drainage continuity and predisposition to clearance inefficiency	[65]
Perivascular phase mismatch	Venous–interstitial fluctuations of 0.15–0.3 mmHg with 17–42° delay relative to CSF/interstitial coupling	Slow-flow corridors (~60–160 µm) and partial decoupling from arterial pulsatility	Prolonged solute residence (2.8–4.3×) and formation of localized retention zones	[12]
Astroglial adaptive response	Endfoot expansion (5–8%), transient cytoskeletal softening, AQP4 polarization loss (6–9%)	Reduced local perivascular water flux and altered glymphatic efficiency	Focal impairment of CSF–ISF exchange and remodeling of astroglial microenvironments	[66]
Barrier stress response	Circumferential wall stress increase (5–7%), nanoscale junctional discontinuity, episodic extravasation	Perivascular entry of plasma-derived solutes and mild ionic shifts (K^+^ + 0.2–0.5 mM)	Microdomain ionic instability and possible effects on nearby axonal conduction	[67]
Axonal conduction sensitivity	Local ionic disequilibrium, metabolic strain, transient Na^+^/K^+^ homeostatic load	Small conduction delays (0.4–0.8 ms) and reduced temporal precision	Drift in tract-level synchrony and weaker timing coordination across long-range pathways	[68]
Metabolic stress patterning	Retention of 3–50 kDa solutes and oxidative marker increase (5–12%) along unstable venous territories	Heterogeneous oxidative microdomains and greater mitochondrial burden	Selective vulnerability of deep white matter and diminished metabolic resilience	[69]
Network-level variability	Patchy clearance deficits, conduction timing noise, reduced low-frequency coherence (4–9%)	Phase instability and weaker slow-frequency coupling	Impaired large-scale coordination and reduced sensory–cognitive integration	[70]
Early systems vulnerability	Accumulation of local vortex burden, persistent clearance inefficiency, metabolic microstress	Progressive decline in network robustness and greater perturbation sensitivity	Putative substrate for early neurological vulnerability, including sleep disruption, cognitive variability, and seizure susceptibility	[71]

**Table 2:** Each therapeutic vector is mapped to a precise nanomechanical leverage point—torsional stiffness tuning, hysteresis correction, torque-grid stabilization, membrane-order enhancement, viscosity shaping, curvature normalization, or mechanotransductive recalibration.

Therapeutic Vector	Primary Nanomechanical Target	Mechanistic Leverage Point	Quantitative Correction Window	Predicted Functional Benefit	References
Torsional-Load Buffers	Spectrin–actin hinge junction (α20–βI)	Raises torsional stiffness; stabilizes helix angle; suppresses torque-amplified instabilities	↑ hinge modulus 11–15%; K_D 180–290 nM	Restores monotonic shear-field alignment; prevents early siphon bifurcation	[130]
Spectrin-Conformation Correctors	βII-spectrin repeat 8–10	Normalizes force–extension hysteresis; reduces axial elongation variance	Variance ↓ 9% → 3–5%	Lowers microvortex persistence; stabilizes near-wall flow gradients	[131]
Rotational Gatekeepers	α-actinin–spectrin torque-distribution grid	Stabilizes duty-cycle of force-gating nodes; prevents uneven torsional loading	Maintains torque spacing 22–29 nm	Suppresses retropropagating pressure pulses; enhances siphon continuity	[132]
Lipid-Grid Enhancers	Sphingomyelin–cholesterol nanotiles	Increases in-plane order; reduces enthalpy fluctuations	S-order ↑ 0.05–0.09; enthalpy ↓ 14–18%	Strengthens lamellar rigidity; prevents curvature-driven wave distortion	[133]
Nanoviscosity Modifiers	Unsaturated PC nanodomains	Tunes damping viscosity; prevents lamellar overshoot	Viscosity stabilized at 0.44–0.56 Pa·s	Enhances oscillation damping; protects lamellar waveform fidelity	[134]
Curvature-Field Normalizers	Lysophospholipid/PE curvature foci	Reduces curvature gradient variance; normalizes bending propagation	Curvature variance ↓ 21–29%	Prevents asymmetric bending waves; improves perivascular pressure coupling	[135]
Microcassette Harmonizers	Mechanosensitive channel lipid–protein interfaces	Increases tension threshold; reduces flicker and noise activation	Threshold ↑ 6–10%; flicker ↓ 18–26%	Prevents noise-driven oscillatory perturbations; improves oscillatory coherence	[136]
Calcium-Codon Regulators	Ca^2+^ microdomain generators (ER–membrane nanojunctions)	Tightens Ca^2+^ codon duration; shrinks diffusion radius	Codon duration 8–11 → 6–8 ms; radius 450 → ~250 nm	Reduces hinge hypercontraction; sharpens mechanochemical phase alignment	[137]
Oscillatory-State Rebalancers	Annexin–PI(4,5)P_2_ stiffness integrator	Maintains membrane stiffness tolerance; prevents resonance drift	Stiffness drift ↓ 35–41%	Sustains long-timescale oscillatory stability; avoids chaotic attractor entry	[138]
Resonance-Space Sculptors	Coupled hinge–lamella energy basins	Reshapes resonance potential; stabilizes broad oscillatory modes	Stabilizes modes 40–110 Hz	Enhances resistance to chaotic transitions; expands stable oscillatory corridor	[139]
Waveform Prediction Inhibitors	Hinge–lamella phase-lock coupling	Reduces higher-order coupling; limits harmonic overflow	Coupling coefficient 0.43–0.58 → 0.36–0.44	Prevents burst-stacking; promotes linear oscillatory propagation	[140]
Adaptive Biomechanical Correctors	Mechanotransductive stiffness feedback loops	Applies real-time stiffness compensation under shear load	Correction 2–4% for shear > 8%	Maintains siphon continuity under hydrodynamic stress; stabilizes oscillation morphology	[141]

## Data Availability

No new data were created or analyzed in this study. Data sharing is not applicable to this article.
